# Advances in the Detection of Emerging Tree Diseases by Measurements of VOCs and *HSP*s Gene Expression, Application to Ash Dieback Caused by *Hymenoscyphus fraxineus*

**DOI:** 10.3390/pathogens10111359

**Published:** 2021-10-21

**Authors:** Piotr Borowik, Tomasz Oszako, Tadeusz Malewski, Zuzanna Zwierzyńska, Leszek Adamowicz, Rafał Tarakowski, Sławomir Ślusarski, Justyna Anna Nowakowska

**Affiliations:** 1 Faculty of Physics, Warsaw University of Technology, 00-662 Warszawa, Poland; pborow@poczta.onet.pl (P.B.); Rafal.Tarakowski@pw.edu.pl (R.T.); 2 Forest Protection Department, Forest Research Institute, 05-090 Sękocin Stary, Poland; T.Oszako@ibles.waw.pl (T.O.); S.Slusarski@ibles.waw.pl (S.Ś.); 3 Department of Molecular and Biometric Techniques, Museum and Institute of Zoology, 00-679 Warsaw, Poland; tmalewski@miiz.waw.pl; 4 Faculty of Biology and Environmental Sciences, Institute of Biological Sciences, Cardinal Stefan Wyszynski University in Warsaw, 01-938 Warsaw, Poland; z.zwierzynska@gmail.com (Z.Z.); j.nowakowska@uksw.edu.pl (J.A.N.)

**Keywords:** ash, dieback, root rot, e-nose, heat shock protein, forest protection, *Hymenoscyphus fraxineus*, *Armillaria gallica*

## Abstract

Ash shoot dieback has now spread throughout Europe. It is caused by an interaction between fungi that attack shoots (*Hymenoscyphus fraxineus*) and roots (*Armillaria* spp., in our case *Armillaria gallica*). While detection of the pathogen is relatively easy when disease symptoms are present, it is virtually impossible when the infestation is latent. Such situations occur in nurseries when seedlings become infected (the spores are carried by the wind several dozen miles). The diseases are masked by pesticides, fertilisers, and adequate irrigation to protect the plants. Root rot that develops in the soil is also difficult to detect. Currently, there is a lack of equipment that can detect root rot pathogens without digging up root systems, which risks damaging trees. For this reason, the use of an electronic nose to detect pathogens in infected tissue of ash trees grown in pots and inoculated with the above fungi was attempted. Disease symptoms were detected in all ash trees exposed to natural infection (via spores) in the forest. The electronic nose was able to detect the pathogens (compared to the control). Detection of the pathogens in seedlings will enable foresters to remove diseased trees and prevent the path from nursery to forest plantations by such selection.

## 1. Introduction

*Hymenoscyphus fraxineus* is an alien invasive pathogen [1] that has spread rapidly across Europe in the last 20 years, causing massive declines in ash trees of all ages [2,3]. The presence of *H. fraxineus* was recorded as early as 1990 in East Asia (Japan, Korea, Northeast China), where it occurs as a harmless saprotroph on *Fraxinus mandshurica* and *Fraxinus rhynchophylla*, suggesting that European ash dieback has an East Asian origin. Symptoms of the disease include necrosis of leaves and twigs, discolouration of wood, wilting of shoots, and death of twigs, branches, and stems. This damage occurs because after colonising bark and wood tissues, and the fungus secretes a phytotoxin, e.g., viridiol, which causes the death of plant tissues [4]. We hope that an electronic nose (e-nose) will detect this or other volatile compounds. When branches die, the tree sprouts epicormic shoots near the infection site to replace those lost, often giving the tree a bushy appearance. These too may eventually succumb to the disease [5]. Although the disease is easy to detect in its advanced form, there is usually no hope of saving the trees, so it is essential to detect it in nursery stock. The pathogen has likely appeared on ash seedlings in nurseries because it was introduced there. Often nurseries bring in young seedlings to be subcultivated before they are sold. It is also possible that wind-blown ascospores infected the seedlings in the nurseries. There are known cases of other species, e.g., *Phytophthora*, being brought in with river water for plant watering. This route can hardly be ruled out either.

Ash trees grown in nurseries should be pathogen-free, but the pesticides, fertilisers and good water regimes mask the disease. Then seedlings planted in plantations and left alone will die en masse. If e-nose can detect the pathogen in asymptomatic plants already in a nursery, it will help foresters select the suitable propagation material for plantings in the forest.

The roots of weakened ash trees are attacked by a fungus of the genus *Armillaria*. The genus *Armillaria* is distributed worldwide and currently includes over 40 officially described species [6]. These pathogens play a significant role in the death of many trees and stands in orchards, vineyards or ornamental plants in gardens. Indirect interactions (via host) among pathogens *H. fraxineus* and *Armillaria* spp. result in severe shoot damage in crowns, at the base of trunks and rot of roots [1,7,8,9,10]. The conidia of *H. fraxineus* are abundantly produced on freshly infected petioles in autumn and winter [11] whilst the ascospores are produced on fallen leaves next summer. The fungus can survive inside petioles under natural conditions for up to five seasons after leaf fall [12]. The presence of fungal fruit bodies allows the detection of both pathogens with the e-nose in the litter and the infestation of tree roots. In the case of *Armillaria*, the e-nose would then be the only device that can detect pathogens in roots without digging them up in young forest plantations and mature trees. Such an advantage could be used in cities where falling trees from wind because of *Armillaria* damaged roots threaten the health and property of their residents.

The development of e-nose technologies for disease diagnosis began in the biomedical field in the mid-1980s for detecting biotic (microbial) causes of human disease [13]. The use of e-nose devices for disease diagnostic applications has subsequently expanded to plant and animal hosts for numerous analyses that include non-invasive early detection of plant and animal diseases through the invention of new gas sensor device types and disease detection methods with sensor arrays designed and adapted for different chemical classes of volatile organic compounds (VOCs) closely associated with individual diseases [13].

Recent advances in e-nose technologies, derived from numerous types of aroma sensor technologies, have been developed for a variety of applications in the broad areas of agriculture and forestry [14,15]. E-noses have been used in various commercial agricultural industries, including agronomy, biochemical processing, botany, cell culture, plant variety selection, environmental monitoring, horticulture, pesticide detection, plant physiology, and pathology. Applications in chemotaxonomy, stem tracking, wood and paper processing, forest management, forest health protection, and waste management in forestry also exist. Thinking about the potential and expected specificity of e-nose for plant disease diagnosis, one might get the impression that e-nose is used to detect diseased or severely stressed plants, regardless of the source of the stress. In forestry, one can imagine such diagnostics being required in many areas, from selecting seedlings to be planted in forest plantations to selecting trees to be felled as part of silviculture or forest protection (sanitary cuttings). Secondary pests or pathogens easily attack weakened trees left in the forest. Currently, the selection of weakened trees is made intuitively based on the forester’s experience (e.g., selecting so-called weakened trees for felling based on the crown’s appearance). However, when droughts occur (and they are becoming more frequent and longer), many trees that belong to the co-dominant or even dominant group according to the Kraft classification are weakened. In this case, the changes detected by e-nose at the level of volatile secondary metabolites would be beneficial to decide which trees should be considered for removal in the context of clearing or thinning to improve the growing conditions for the remaining trees. In this way, we can use the natural processes of plants to produce volatiles (secondary metabolites) for defence, such as the well-known caffeine in coffee or nicotine in tobacco. We are currently looking for these substances in trees using gas chromatography with a GC-MS mass spectrophotometer. They belong to the phenolic compounds, sterols, and terpenes [15,16].

An important limitation of electronic nose applications should be acknowledged compared to classical chemical analytical methods such as GC-MS. Unlike the former, the measurements performed by e-noses cannot provide objective information about the chemical components present in the measured samples. The analysis of the captured signals applies machine learning pattern recognition methods, which often act as a black box and cannot explain the classification. Different sample types of different chemical compositions may cause similar patterns in the sensor’s responses, making it impossible to differentiate between the studied species. That is similar to human experience when various odours can give similar impressions. Another drawback of the approach of applications of the e-nose measurement combined with the machine learning techniques is the difficulty in detecting new patterns in data when new odours are not present in the training phase of the experiment.

Nevertheless, artificial intelligent noses have also been used as rapid and non-invasive tools for diagnosing insects and diseases affecting vegetables and fruit trees [17] with particular focus on bacterial, fungal and viral infections as well insect damage. Volatile organic compounds emitted by plants provide helpful information about plant growth, defence, and health, offering the possibility of non-invasive monitoring of plant health [15]. Compared to the traditional gas chromatography-mass spectrometry (GC-MS) technique, e-noses are non-invasive and can be a fast, low-cost option for various applications. However, the use of e-noses for plant pest diagnosis is still under development. There are challenges related to sensor performance, sampling and detection in open areas, and scaling of measurements. This paper tests feasibility of using PEN3 e-nose for early detection of ash fungal pathogens while investigating their interactions and ash seedlings. It includes a comprehensive comparison between three seedling treatments, i.e. natural *H. fraxineus* and artificial *A. gallica* inoculations and phosphite fertiliser, which will allow us to discriminate above fungi in the soil, as well as assess the health status of ash.

One of the plant adaptation mechanisms is the ability to produce different VOCs [18], which are involved in response to biotic stress such as herbivory [19], mechanical damage [20], pathogens [21]. As an indicator of stress level in plants, we used *Hsp*s and their primary regulator-heat shock factor gene (*Hstf*) expression measurement. Stress caused by abiotic factors can add up or have a synergistic effect. In addition, stress factors, according to the so-called chain disease concept, may follow one another (when one ends, another begins), or, as in Manion’s spiral disease theory [22], stress factors may occur simultaneously. Manion divides them into predisposing (acting for a long time like drought), initiating (causing damage like *H. fraxineus*) and contributing (e.g., *Armillaria*). The damage caused by *Armillaria* could be largely due to a reduced ability to absorb water. This observation supports Manion’s theory that contributing factors in the forest ecosystem (at a particular place and time) directly cause the tree or stand death. They benefit from the weakening of trees by predisposing and inciting (triggering) factors. In the case of ash dieback, the most important factors act together: predisposing factors such as climate change (and the resulting drought), *H. fraxineus* (inciting) and *Armillaria* (contributing).

Heat shock proteins (*Hsps*) play an extensive role in many cellular processes, giving them a general role in tolerating different environmental stress treatments. Maintaining proteins in their functional conformation and preventing aggregation of non-native proteins are particularly important for cell survival under stress [23,24,25]. Under stress, these stress-responsive biomolecules act as molecular chaperones through up- or down-regulation [26]. Stress has a significant impact on the expression of *Hsp* [26,27,28]. The expression of heat shock protein genes in ash has not yet been investigated. To assess the molecular response to plant damage caused by phytopathogens, we examined the expression of three selected *Hsp* genes, *Hsp17, Hsp70, Hsp90* and one of their regulator-heat shock transcription factor (*Hstf*). Recently the only available data for ash are *Hsp17, Hsp70, Hsp90* heat shock genes and one of their regulators-heat shock transcription factor (*Hstf*). All the genes were used to evaluate stress level after *A. gallica* and Actifos treatment of ash.

In summary, the main objective of this study was to determine the feasibility of detecting ash pathogens using e-nose, using the example of a fungus attacking the shoots of *H. fraxineus* (needed for seedling selection in nurseries) and the root systems of *A. gallica* (needed for older trees that may be at risk), and the study of *Hsp* gene expression was to show the response of ash trees to infection.

## 2. Materials and Methods

### 2.1. Experimental Design

#### 2.1.1. Preparation of the Plant Material

The 3-year-old trees *F. excelsior* in the study were grown in 10-L pots (with garden soil of pH 5.5–6.5) and originated from seeds collected from trees growing along roadsides, which did not show dieback symptoms common in forest stands. After spending a year in the forests, they were grown in a greenhouse (temperature range there between 1 °C (winter) to 30 °C (summer), and the photoperiod was the same as in nature. The study included 48 ash seedlings divided into five treatments: C—control, A—seedlings implemented with Actifos, G—seedlings inoculated with the pathogen *Armillaria gallica* and AG—seedlings treated with Actifos and inoculated with *A. gallica*. We had 8 samples of the Control (C) category and 10 samples of each other category as it is presented in Table 1.

**Figure 1:**
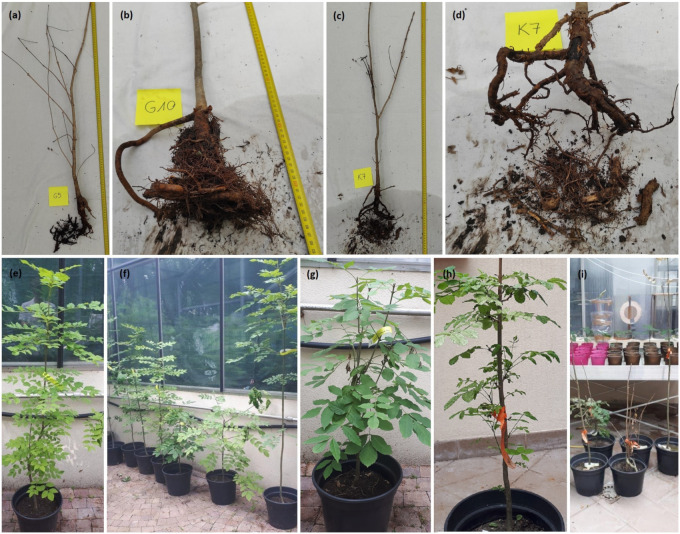
Examples of potted ash trees (**a**,**b**) dead and diseased ash trees, both with roots damaged by *A. gallica*; (**c**,**d**) control ash trees exposed to infestation by *H. fraxineus* in the forest; (**e**–**g**) control ash trees left in the greenhouse (isolated) and showing no disease symptoms; (**h**,**i**) ash trees treated with Actifos phosphate fertilizer.

The isolated control (I) variant included plants grown in a separate box under natural conditions (they were not directly exposed to foliar infestation by conidia and ascospores). In contrast, all other plants listed were brought into the forest and placed under the canopy of mature ash trees so that they were exposed to the natural pressure of *Hymenoscyphus fraxineus* inoculum throughout 2016. The selected photographs of plants prepared in our experiment are presented in Figure 1.

#### 2.1.2. Preparation of the Fungal Inoculum

Inoculation of plants with *Armillaria gallica* Marxm. & Romagn. was carried out with pieces of hazelwood *Corylus avellana* L. For preparation, these were autoclaved (120 °C, 30 min) after cutting into sections about 10 cm long and placed in metal boxes containing pure fungal culture obtained from a mixed forest. After 6 months, when the wood was completely colonised and the rhizomorphs began to grow outward, they were placed next to the roots in the soil of potted plants depth of approximately 2 cm. The plants’ treatments (A and AG) with ammonium phosphate (Actifos) were applied once in May 2016. Seedlings were treated with a 0.6% aqueous solution of Actifos ((NH${}_{4}$)${}_{2}$HPO${}_{3}$ from Agropak, Poland). Its full chemical composition is as follows: nitrogen—10.2%, boron—0.02%. copper—0.008%, iron—0.06%, manganese—0.04%, molybdenum—0.004%, zinc—0.02%. Actifos spraying aimed to induce natural resistance in the plants and protect them from natural infections. The isolated control (I) was free from all these treatments and grew in a separate box in the greenhouse under natural conditions throughout the study period.

#### 2.1.3. Detection of *Hymenoscyphus fraxineus* in Ash Tissues

To confirm successful natural inoculation (via spores) and damage caused to shoots (one per plant was tested), we used the molecular technique described by King and Webber [29]. The primers Hym_F ′5-GCGAATGAATATGGGCTTACA-3′ and Hf_R ′5-GCATAGCGTGGCTCTCTGG 3′ were used to detect *H. fraxineus*. Polymerase Chain Reaction (PCR) was performed with a total sample volume of 20 μL in a Veriti 96 well AB thermal cycler (Applied Biosystems, California, USA). Each sample contained: 3 μL of genomic DNA, 10 μL of RedTaq PCR ReadyMix (Sigma-Aldrich, Milwaukee, WI, USA), 1 μL of each (forward and reverse) 5 μL primer, and sterile water to a final volume of 20 μL according to King and Webber [29]. PCR reaction conditions were: initial denaturation 95 °C for 3 min, 35 cycles of 95 °C for 1 min, 57 °C for 1 min and 72 °C for 1 min; a final extension of 72 °C for 5 min. PCR products were resolved on 2% agarose gels.

#### 2.1.4. Plant Biomass Assessment

The number of shoots that died at the end of the experiment was counted and compared between all treatments. At the end of the experiment, all plants were removed from their pots and roots were cleaned with tap water. Later, roots were placed in a Termaks series 2000 dryer (KJ Auktion, Aalborg, Denmark) and dried at a temperature of 64.9 °C until total evaporation of water. The weight of roots biomass was then measured on RADWAG WTC 2000 scale (Jawag, Jarosław, Poland) in three replicates per single plant. To validate all dry biomass measurements, the Student *t*-test was performed in the Statistica program ver. 12.0 (StatSoft, Tulsa, OK, USA), for α = 0.05. Data with *p*-values of <0.05 were considered statistically significant.

### 2.2. Electronic Nose

#### 2.2.1. PEN3 Electronic Nose

**Figure 2:**
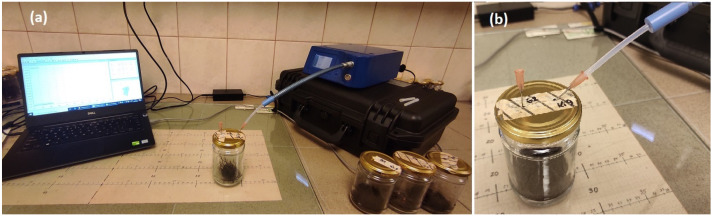
(**a**)—Measurement setup using PEN3 electronic nose. (**b**)—Highlighted jar with soil sample during measurement procedure.

The electronic nose measurements in the reported experiments were performed with a commercially available device, PEN3 (Airsense Analytics GmbH, Schwerin, Germany). The measurement setup used in our experiment is presented in Figure 2. The PEN3 consists of a gas sucking and sampling installation, a detector unit containing the sensor array composed of 10 metal oxide semiconductor (MOS) type chemical sensors, and software for data collection and pattern recognition. The device sensors, listed in Table 2 are working at high temperatures (150–500 °C) and respond to the broad range of chemical compounds.

**Figure 3:**
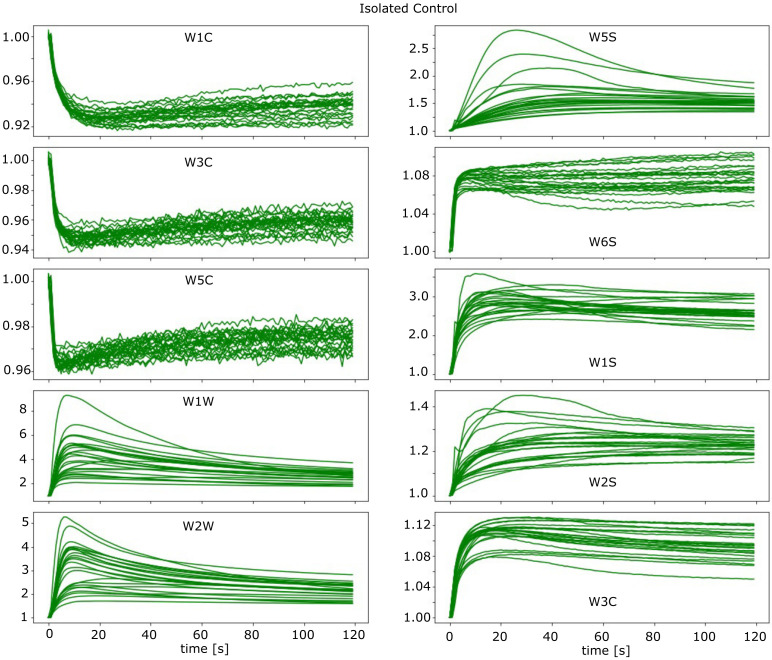
Sensors responses as conductance normalized by the baseline value (G/G${}_{0}$), for measurements of roots samples of Isolated Control category.

As is recommended by the manufacturer, the PEN3 device has been pre-warmed for at least 10 min before a series of measurements. Before measuring each sample, the clean air, filtered by the activated carbon, was blown in reverse through the electronic nose to clean the sensor array and the gas transmission tubes. During the measurement phase, the headspace gas of the sample was sucked into the sensor chamber at a constant flow rate. The gas was sampled using a tube connected to a needle. The measurement time was set to 120 s, and sensors response values were acquired every second using the producer’s software. The examples of collected response curves of the electronic nose are presented in Figure 3, where the conductance of the sensor normalised by the baseline value (G/G${}_{0}$) is plotted.

The baseline value was measured when the sensors were exposed to clean air conditions at the beginning of the measurement. Details of operation conditions of the electronic nose are listed in Table 3.

#### 2.2.2. Taking Measurements with E-Nose

The present study used a portable electronic nose PEN3 based on a standard metal oxide gas matrix. Three root samples (and rhizomorphs in G and AG variants) and three rhizospheric soil samples were collected from each ash seedling growing in the IBL greenhouse. The material was collected in jars and weighed (roots about 5 g, soil about 50 g). After sealing the samples in the jars, they were placed in a room with a constant temperature of 25 °C and humidity of 60%. The samples were measured 1–3 h after collection. For this purpose, the lids of the jars were previously drilled so that they had 2 holes, which were sealed with a special high-temperature resistant film for the time of autoclaving, then these places were pricked with needles for the time of measurement (120 s). The measurement setup used in our experiment is presented in Figure 2.

The samples preparation and e-nose measurements were performed during several days in randomised order. First of all, at the beginning of the day of the measurements, one seeding from each of the studied variants was randomly selected. Then, samples for measurements were prepared, and from each seeding, 3 samples of roots and soils were collected. The samples were measured in random order, and the randomisation was performed using Microsoft Excel random number generator.

### 2.3. Electronic Nose Data Analysis Techniques

We applied two commonly used statistical methods to analyse data collected by the measurement performed by the PEN3 electronic nose. First of all, we extracted from the sensors’ response curves several types of features describing their shapes to visualise the data, which is helpful to understand appearing patterns. For that purpose, we applied the Principal Component Analysis method. However, the primary type of data analysis was to build several machine learning classification models to evaluate the possibility of differentiating between the considered categories of the samples. All analysis and visualisation of electronic nose data presented in this report were conducted using Python 3.7 language codes, using statistical analysis methods from the scikit-learn module [30].

#### 2.3.1. Classification Models

The main goal of odour measurements by electronic nose device is to differentiate between studied sample categories by finding patterns in collected signals and applying statistical machine learning methods. The electronic nose device does not analyse the chemical composition of gases as it is possible by analytical methods such as gas chromatography-mass spectrometry.

Machine learning methods can be applied to create statistical classification models to discriminate between the studied samples using the data collected by the electronic nose measurements [31,32,33]. In this task, we follow a well-established methodology.

Before the model training process, the modelling features are extracted from the response curves, which allow reducing the dimensionality of the problem [34,35]. In the performed experiments, one sample measurement produces 1200 data entities. This number equals the number of sensors multiplied by the number of reads of sensor conductance magnitude performed during 2 min, with a 1-second interval. The first type of such feature group is the sensor’s response magnitude at the characteristic moments, such as at the end of the measurement, at max/min of the response, or the indicated moment elapsed from the beginning of the sensor’s exposure the odour [36,37,38]. The following modelling features are other essential characteristics of the response curve: average (equivalent to the integral/area under the curve), standard deviation, skewness, and kurtosis. In addition, the basic statistics such as max, min, std, mean, skewness, kurtosis, calculated from the response curve derivative [39,40,41], after smoothing by the exponential moving average method. The following features are characteristic times, such as the time to reach 10%, 25%, 50%, 90% of the sensor response range, and time to reach max/min of the curve derivative.

As the first step of the model training procedure, the used dataset of features was split into two parts: the testing subset, used later only for independent estimation of the model performance, and the training subset used for model training and selection. For this task, we applied group splitting, which assured that all data collected from a given tree sample had been assigned to testing or training datasets. Our analysis for the testing dataset took apart data from randomly selected two trees of each category.

In the next step, the series of classification models were trained, and the most important features were selected using the recursive forward selection algorithm [35]. For that purpose, the training part of the dataset was again split into two parts: the first one was used to estimate the model parameters, and the second one to validate the model performance and select between competitive models using the accuracy measure. The validation was performed ten times in a loop for various random selections of the training split. We also applied the random group shuffling method for this data split, assuring that data collected by the measurements of a given tree were used either to model training or validation. For the validation part, we took apart data collected from two trees. As a machine learning modelling technique, we used Logistic Regression.

When the best-performing model has been selected, the data from the testing dataset were scored, and the model performance statistics were calculated.

The described above procedure has been repeated as the shuffling cross-validation (CV) procedure when testing and training subsets data collected from different trees have been assigned. We repeated the CV loop 30 times for random selection of these subsets groups. The estimates of the model performance statistics have been averaged over the CV results. We used accuracy to evaluate the model performance, defined as the ratio of correctly classified samples to all samples.

#### 2.3.2. Principal Component Analysis

In our studies, we performed the commonly used statistical technique of Principal Component Analysis (PCA) to transform the input data to lower-dimensional space, which helps to give intuitive insight and understand the patterns appearing in the distribution of data for the studied case. The PCA transformation has an intuitive interpretation as the rotation of the coordinate system, giving the new coordination in the order of quantity of the captured variability of the dataset. As the input for the PCA transformation, we used the set of features extracted from the sensor’s response. The set of features selected by the machine learning classification model was used as input for the PCA transformations. Since the input features have different ranges and represent non-comparable quantities, we used the initial normalisation of the input dataset to equal variance.

In our analysis, we build binary classification models for classification between pairs of the studied categories. For that reason, data visualisation is also performed in pairs of sample categories. We chose the models containing five features.

### 2.4. Heat Shock Protein and Heat Shock Transcription Factor Gene Expression Analysis

To investigate the molecular response of ash to stress, we analysed expression heat shock protein genes. For designing of primers we used sequences of three heat shock protein genes (*Hsp17*, *Hsp70* and *Hsp90*) as well as heat shock transcription factor (*Hstf*) of *Fraxinus pennsylvanica* from Hardwood Genomics Project database https://hardwoodgenomics.org (accessed on 1 June 2021). Currently, sequences are available for above mentioned heat shock proteins genes only. Unfortunately, there are no available sequence data for heat shock protein genes for *F.excelsior*. Tubulin (*Tub*) gene was used as a reporter gene in our experiment. Total RNA was extracted from leaves of *F.excelsior* using Plant RNA Mini Kit (Syngen Biotech, Wrocław, Poland), following the manufacturer’s protocol. The total RNA extracted and its purification from protein and polysaccharides were determined using a NanoDrop 2000 spectrophotometer (Thermo Fisher Scientific, Waltham, MA, USA). RNA integrity was checked electrophoretically in a 1.5% agarose gel stained with ethidium bromide. Only samples that met both quality and integrity requirements were used in subsequent experiments. Three high-quality RNA samples (i.e., biological replicates) were obtained for each condition. The reverse transformation was performed using the GoScriptTM Reverse Transcription System (Promega GmbH, Karlsruhe, Germany) according to the manufacturer’s instructions. Primers for analysis of *Hsps* gene expression were designed using Primer-BLAST [42] under default parameters. Real-time PCR reactions were performed in 20 μL volume: 10 μL 2× qPCR SYBR Master Mix (Sigma Aldrich, Milwaukee, WI, USA), 2 μL cDNA, 2 μL each primer (5 μM forward and reverse) (Genomed, Warsaw, Poland), 6 μL H${}_{2}$O. Thermocycling conditions consisted of the initial denaturation step at 95 °C for 3 min and 40 cycles at 95 °C for 10 s, annealing at 55 °C for 20 s, and elongation at 72 °C for 20 s. Real-time PCRs were performed in the 7500 Real-Time PCR system (ThermoFisher Scientific, Waltham, MA, USA). Based on obtained Ct values, the 2Ct method was used to calculate the relative ratio of *Hsps*’ expression, but the correct amplification efficiency was used instead of the value 2 [43]. We used a noise-resistant iterative nonlinear regression algorithm (Real-time PCR miner; www.miner.ewindup.info, accessed on 1 June 2021) to determine the efficiency of the PCR reaction [44].

### 2.5. Detection of *H. fraxineus* and *A. gallica* in Ash Tissues

The PCR reaction confirmed the successful infection of all ash shoots with *Hymenoscyphus fraxineus*, indicating that the amount of fungal inoculum in the air was abundant and sufficient to cause natural infection and the development of dieback symptoms. We also found successful inoculation of root systems with hazel canes colonised by *A. gallica*. All root systems were damaged to a greater or lesser extent by the fungus (Table 4).

## 3. Results

### 3.1. Number of Shoots and Weight of Roots Biomass

At the end of the experiment, we counted the number of dead shoots and weighted the dry biomass to evaluate the condition of the measured samples. The descriptive statistics and *p*-value obtained from these measures are presented in Table 4.

The shoots affected by *H. fraxineus* died in all the experimental variants. On average, the highest number of dead shoots was observed in variant A (7.3) and the lowest in I (3.1). Variant G had a similar average value for the number of dead shoots as variant A (7.1). The seedlings of C and AG had an average of 4.4 and 5.4 dead shoots, respectively.

Spraying shoots with Actifos (A) did not affect protection against the pathogen, and the number of dead shoots was similar in ash trees with roots infected by *A. gallica* (G). Seedlings not inoculated with *A. gallica* (C) and those inoculated with *A. gallica* but treated with Actifos at the same time (AG) showed similar infestation but at a lower level. Only ash trees that were not exposed to the fungal spore inoculum (isolated) had statistically significantly fewer dead shoots, indicating that the amount of inoculum in the forest is still high, and it would be difficult to establish a healthy ash stand nowadays.

The lowest average dry root weight was recorded in variant A (35.6 g) and the highest in variant I (603.5 g). The pathogen *A. gallica* (G) caused a statistically significant reduction in root biomass, which rotted as in the forest. The *p*-value for the Kruskal-Wallis test by ranks is presented in Table 4. A single spray application of Actifos (AG) almost doubled root biomass (compared to A), indicating the benefits of this treatment for ash trees, probably due to stimulation of natural immune processes. However, Actifos (A) itself hurt root development, which could be due to both its toxic effect (and thus stimulation of immune processes) and the large number of shoots infected with *H. fraxineus* (which prevented the induction of resistance due to lack of sufficient assimilates). This observation is confirmed by other observations on ash trees that were not inoculated with *Armillaria* but were exposed to the natural spore pressure of *H. fraxineus* in the forest. In this case (C), the number of dead shoots was similar (reducing resistance), but the physical absence of the root-degrading fungus almost doubled their dry biomass compared to (A). The dry biomass of isolated ash roots (I) was several times higher than other treatment options. Both the dead shoots and the roots testify to the effectiveness of the fungal inoculation methods (artificial and natural), so we could detect them in diseased ash trees using an electronic nose.

### 3.2. Electronic Nose Sensor Responses

The results of the measurements by the PEN3 electronic nose are values or sensors conductivity as a function of time. These values are unitless as they are normalised by the baseline sensor response to the clean air conditions, measured just before the sensor’s exposure to the measured odour.

In Figure 3 we present the obtained results of measurements of odours of ash roots of Isolated Control samples. As one can notice in this figure, there is significant variability in the results from measurements of various samples. This effect is due to the variability of individual trees and other sources of random variabilities, such as environmental conditions and internal operations of MOS sensors. In other research by the same type of electronic nose device, similar issues of repeatability of measurements results are reported [36,45]. We present the results obtained only from one of the measured sample types, which does not overload this figure. Presentation of all measurements on one single figure in the considered case could not distinguish any patterns just by visualisation. Similar figures for other types of samples are presented in the Supplementary Materials.

**Figure 4:**
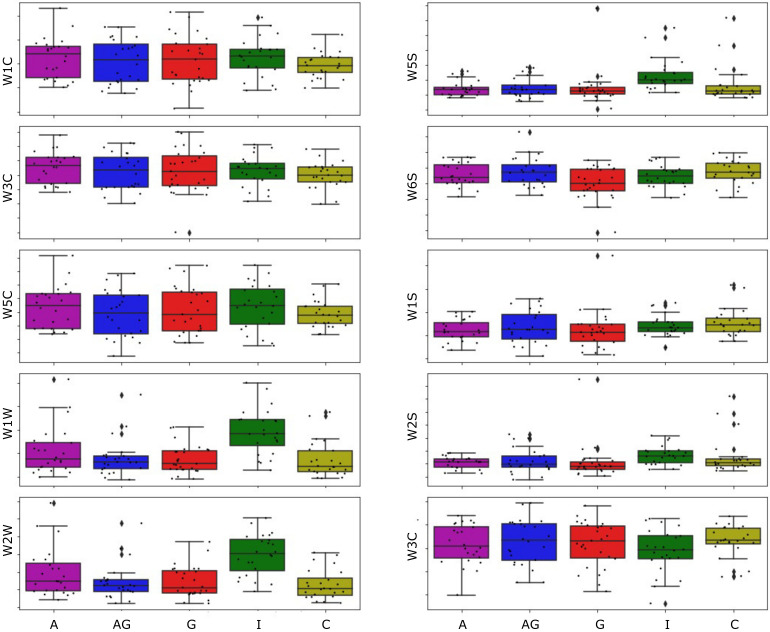
Distribution of modelling feature extracted from the sensor response characteristics (integral/sum of the response data) for measurements of roots samples. The exact value of the *y*-axis for various sensors has no physical interpretation, thus is presented only in arbitrary units. The studied categories of samples: Actifos (A), Actifos+*A. gallica* (AG), *A. gallica* (G), Isolated Control (I), Control (C) are plotted in the *x*-axis. In the box-plots, the horizontal line inside the box represents the sample median, the box area spans from the 1st to the 3rd quantile, the whiskers span from Q1−1.5 * IQR to Q3 + 1.5 * IQR (IQR—interquantile range). This plot overlays all data points presented by dots, allowing us to see outliers and the whole data distribution.

As we already mentioned, it is common to represent the sensor response curves by a smaller number of features describing the shapes of the curves for analytical purposes. It may be instructive to observe the distribution of measurement points for one of such features and notice differences between the studied categories. In Figure 4 we draw a box-plot chart for the area under the curve feature, for all used sensors, with grouping by studied categories of the samples.

Several important observations can be noticed after examining the plotted data. In this figure, we present data collected in the experiment when samples of roots were measured. In the case of measurements of soil samples, we were not able to notice any patterns. These data for soil measurements are presented in the figure available in the Supplementary Materials.

First of all, there is no apparent difference pattern between Isolated Control (I) samples and other types of samples. It is most noticeable when we look at measurements performed by sensors W1W (sulfur-organic), W2W (sulfur-chlorine), and W5S (broad-range), but also a slight difference can be noticed for the sensor W2S (broad-alcohol). From Figure 4 it could also be noted that the box-plot for Isolated Control is higher than for the other categories. As we see from Figure 3 these sensors in the presence of the measured gases react by an increase of the measured conductance value. That means that also the displayed in Figure 4 feature increases for higher sensor reaction. From that observation, we can conclude that odour components cause the noticed difference in higher quantities in Isolated Control samples than other sample categories.

In this figure, we can also observe that there is no pattern indicating the possibility of differentiation samples infected by the *A. gallica* (G and AG) and not infected samples (A) and (C), at least for the considered modelling feature. The data distributions for (G) and (AG) samples strongly overlap with distributions for (A) and (C) samples.

### 3.3. Classification Models

In our studies, we built several machine learning classification models with various targets. First of all, we tried to distinguish between the samples infected by the *A. gallica* fungus and other samples. We failed in these attempts and could not train a model that could be efficient in such a task.

Our subsequent analysis focused on a more detailed examination and trained series of binary target classification models for differentiation between pairs of studied categories of samples. In this task, we proceeded with a few further refinements of the models to improve their performance.

In the beginning, we tried to use the sensors’ response values as modelling features reached the final moment of observation. These features have not led us to satisfactory classification performance. Thus, we tested another approach to model building various complex features describing the sensors response curves described in the Materials and Methods section. We noticed an improvement in the modelling performance but also had some more observations that allowed further improvements.

One observation that was already mentioned after examination of Figure 4 was that the most helpful information for differentiation between studied cases is contained in signals from sensors W1W, W2W, and W5S. This observation was also confirmed by the selection made by the features selection algorithm, as almost only features related to these sensors were used in the models. Thus, we trained a new set of models. Only data obtained by these sensors were used and noticed that the classification accuracy has not degraded compared to the previous attempt. The following observation from examining the modelling features selected by the best performing models was that the most important features are often related to sensors’ behaviour at the beginning of the response curve.

**Figure 5:**
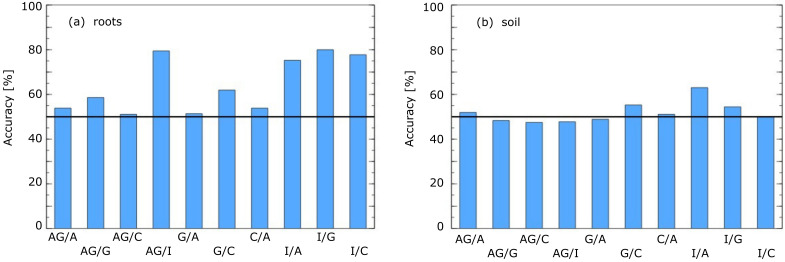
Accuracy of roots (**a**) and soil (**b**) samples differentiation by machine learning models. On *x*-axis comparison between various pairs samples types is presented: Actifos+*A. gallica* (AG), Actifos (A), *A. gallica* (G), Control (C), Isolated Control (I). The horizontal line indicates the performance of the baseline model of random selection.

After that observation, we decided to simplify our approach and use only the raw data collected during the first 10 s of the measurement as modelling features. We prepared a new dataset containing as modelling features the sensors response values collected in the 1-second intervals and the features calculated as proportions of values from pairs of sensors and difference between such values. We had not used the raw data values collected by the electronic nose for all of these features but the values after smoothing the response curves by the exponential moving average. Also, for the training of these models, we used the variable selection procedure. The final results are presented in Figure 5.

As we can notice, for the measurements of roots samples, the accuracy of differentiation between Isolated Control and all other categories of studied samples is 74–78%. The accuracy of differentiation between Control and *A. gallica* samples is about 58%, but as we already admitted, in our opinion, such a value close to a random model is instead saved to treat it as a negative result.

In our experiment, we also tried a more appealing task, testing the possibility of differentiation of the infected samples by examining the odour emitted by the roots and odours of soil samples. Success in it would give hope of detection of fungi infection by non-damaging methods. Unfortunately, we had not observed such a possibility and need to report negative results. This insufficiency is presented in Figure 5, where, as one can notice, the accuracy of the models are very close to the random selection. There is slightly better accuracy for the case of differentiation between Isolated Control and Actifos samples. However, the accuracy performance of about 62% is, in our opinion, not satisfactory for application purposes.

### 3.4. Principal Component Analysis Using Electronic Nose Data

As we discussed above, the results presented in Figure 4 indicate the possibility of differentiation between Isolated Control and other types of studied categories. Such observation was confirmed by building machine learning classification models, which we discuss in the following section. It may also be interesting to visualise the distribution of the studied sample categories after the transformation of the input features by the Principal Component Analysis.

**Figure 6:**
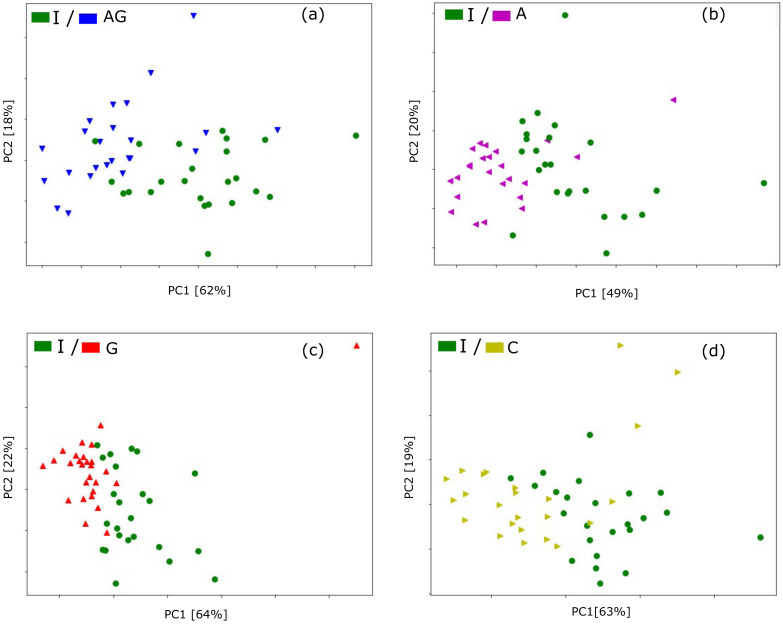
Principal Component Analysis of five most important features selected by the classification models. Measurements of roots samples for differentiation of Isolated Control—I versus (**a**) Actifos+*A. gallica*—AG, (**b**) Actifos—A, (**c**) *A. gallica*—G, (**d**) Control—C, as indicated in the figure panes. Variability captured by the PC is indicated in axis labels.

In Figure 6 we present the two most significant principal components of transformation of these features. These visualisations also confirm a clear distinction between these samples. We present here only the pairs of sample categories, for which, according to the classification modelling, such distinction is achieved.

A remark concerning the PCA transformation of the modelling features should be noted here. As we already explained as the input features, we use the responses of three selected sensors during the first 10 s after their exposure to the studied sample odour. These data are strongly correlated, and the PCA transformation helps to reduce the dimensionality of the dataset. Also, for the PCA transformation, we used the five most important features selected by the classification model. However, since the models are trained in the cross-validation loop, we obtained a set of 30 models for each pair of classified categories. Due to the randomness of the samples, various features were selected. The Figure 6 presents just selected examples.

### 3.5. Heat Shock Protein and Heat Shock Transcription Factor Gene Expression Analysis

**Figure 7:**
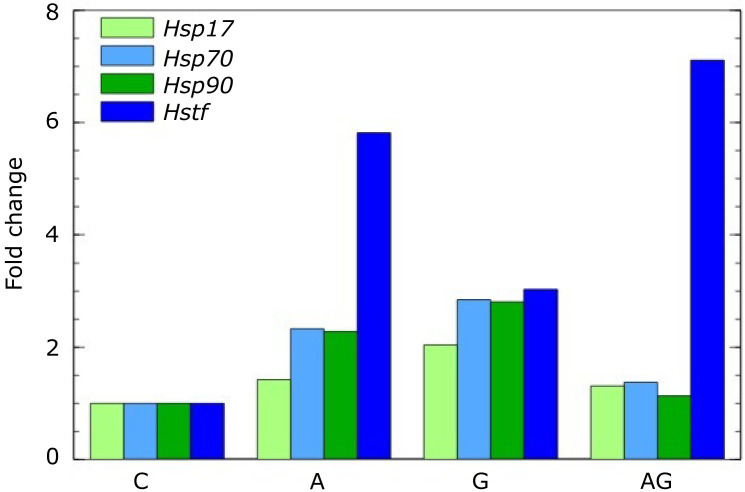
Relative expression level of *Hsp*’s and *Hstf* genes in *Fraxinus excelsior*.

For the analysis of *Hsp*’s and *Hstf* genes corresponding forward and reverse primer pairs were used. Each amplicon was found only in a single peak in melt curves indicating no dimer or multiple products. Analysis of the expression of heat shock protein genes showed that *A. gallica* infection increased *Hsp*17 expression approximatelly 2-fold, *Hsp*70, *Hsp*90 and *Hstf* 2.8–3.0 fold (Figure 7). Similar effect on *Hsp*’s gene expression have actifos but its effect on *Hstf* was higher and increased its expression 5.8-fold. *A. gallica* infection together with actifos treatment have cumulative effect on *Hstf* and increased it expression 7.1-fold. Although the expression of *Hstf* under combined treatment of ash by *A. gallica* and with Actifos increased, the expression of *Hsp*’s genes regulated by this transcription factor not only did not increased but even decreased.

## 4. Discussion

As the title of the article suggests, at this stage, we focus on the problem of ash dieback caused by the fungus *H. fraxineus*. It causes the death of seedlings already in nurseries, and what is worse, some of them end up (symptomless) in forest plantations. Then the cost of eradication is very high if it is possible at all. We, therefore, need a method for early detection of the fungus that allows seedlings to be protected (e.g., chemically) or selected to exclude infected seedlings from further cultivation. Therefore, healthy seedlings (I-isolated) were compared with other seedlings exposed to natural infection with ascospores of the fungus present in the forest. All exposed plants were infected to varying degrees, despite the application of Actifos. This situation frequently occurs in nurseries and later in plantations and was measured by E-nose (Figure 6). It shows that healthy seedlings (I) can be selected from those infected only by *H. fraxineus* (C), or sprayed with Actifos and infected by *H. fraxineus* (A), or by *H. fraxineus* and *A. gallica* (G), or even by *H. fraxineus, A. gallica* and treated with Actifos (AG). The method of damage estimation (number of dead shoots and dry roots biomass) caused by pathogens and their differentiation is presented in Table 1. Note that as healthy seedlings were taken as variant (I), the others were infected by *H. fraxineus* and were additionally inoculated with *A. gallica* (G) or treated with Actifos (A) or arranged both together (AG). The following Figure 4, Figure 5 and Figure 6 show the detection capabilities of these combinations using the electronic nose. These stresses are additive in nature. The additional (relative to *H. fraxineus*) root infection by *A. gallica* resulted in an increase in the average number of dead shoots by almost half (C-4.4, and G-7.1), while the control had an average of only 3.1. Similar relationships were observed between the roots of *H. fraxineus* and *A. gallica* (G), which were heavily damaged (57 g) compared to *H. fraxineus* (C), only 93 g, but the biomass of control plants reached a level 10 times higher (604 g). Wood rot (also caused by *Armillaria* spp. fungi) is a severe fungal disease of trees, requiring clear-cutting of about 100,000 ha of stands annually in Poland (internal reports from forest districts submitted each year to the Forest Research Institute—IBL). These fungi invade trees through roots or open wounds and attack all cell wall components with extracellular digestive enzymes, leading to the destruction of the nutrient-rich sapwood and affecting the strength and stability of the wood. Baietto et al. [46] used a new non-invasive sampling and gas sensor array analysis to detect stem and root rot in living trees successfully. This approach we used to test e-nose on live ash trees to diagnose wood rot disease caused by artificially inoculated *A. gallica* (with pieces of infected hazelwood). Our measurements on seedlings encourage continuing research on adult trees, because to date, assessment of their stability and detection of internal rot have been carried out visually (presence of fruiting bodies and rot at the base of the trunk) or with commercial instruments and methods, which are often invasive (resistograph), time-consuming (Picus echo sounder) and therefore unsuitable for use in forest environments.

Moreover, most conventional instruments do not provide an adequate assessment of decay occurring in the root system. Baietto et al. [46] conducted a long-term research project to develop a novel approach for the internal diagnosis of tree decay by detecting differences in volatile organic compounds released by wood decay-causing fungi and wood from healthy and decayed trees. They (as well as we) tested commercial electronic noses under laboratory conditions, focusing on testing e-noses for their ability to discriminate between different logs and species of wood rot fungi, as well as sapwood from different tree species [46] took the next step in the field, which we also want to do, and tested the diagnostic ability of e-noses to detect differences in odour compounds emitted by healthy and inoculated wood (stem chips and root fragments) in different soil types to assess whether soil odour can affect the ability of e-noses to discriminate between uninfected and diseased root fragments. In the final phase, we would also like to test soil air for the presence of volatile organic compounds released by root-destroying fungi on diseased standing trees.

Early detection of wood rot is critical, especially in high-value stands, because control measures must be initiated long before tree failures result in property damage or injury to citizens. Climate change exacerbates adverse conditions and increases physiological stress on trees, leading to greater susceptibility to attack by pathogenic wood-destroying fungi. Therefore, it is necessary to detect wood rot early, not only in the trunks but especially in the roots that hold the trees in the soil. Detecting fungal root rot on trees is particularly difficult because the conventional detection tools currently used to diagnose wood rot are not applicable below ground level. Portable electronic olfactory systems or electronic noses, now used in many different scientific and industrial fields, have already been tested for early wood rot fungi [47]. Like us, the authors evaluated the accuracy and efficacy of a PEN3 portable electronic nose for discriminating between healthy and decayed root segments artificially inoculated separately with root rot fungal species and incubated in soil under laboratory conditions. The PEN3 electronic nose discriminates between healthy and inoculated root segments and root rot fungi in soil for most host-fungus combinations. In our case, the discriminatory power of the e-nose varied depending on whether the seedlings were isolated (protected) or exposed to foliar infection by *H. fraxineus*.

In conclusion, in ash trees, there is an interaction between shoot damage by *H. fraxineus*, root malnutrition and root rot by *A. gallica*. Root rot caused by *A. gallica* is often the direct cause of trees dying and being blown over by the wind. Therefore, information on whether the tested ash trees are only affected by the shoot mentioned above pathogen or whether their roots are already affected by *A. gallica* root rot can be very helpful in determining other management strategies in both forests and urban green areas. Tree evaluation methods currently used to assess the structural stability of individual trees typically involve visual analysis followed by measurement of the internal strength of the wood using a variety of instruments that are often invasive, expensive, or unsuitable for use in a forest environment. In addition, most conventional instruments do not provide an adequate assessment of decay occurring in the root system. Our subsequent research aims to test the ability to detect differences in volatile organic compounds (VOCs) released by wood decay fungi and wood from healthy and decayed trees. Such studies were previously conducted by Baietto et al. [48]. The authors independently evaluated three e-noses based on different operating technologies and analytical methods (without direct comparison) to determine the ability to detect the onset of decay in artificially inoculated wood. All three e-nose instruments could discriminate between sound and artificially inoculated decayed wood with high precision and reliability.

E-nose technology was recently applied by Cellini et al. [49] to detect various plant diseases and pests with promising results. However, despite numerous advantages such as ease of use, non-destructive nature and bulk sampling, there are also disadvantages such as low sensitivity and specificity compared to microbiological and molecular methods. The above authors also pointed out that electronic nose is crucial in plant disease diagnosis and pest detection, and instrumental and procedural advances in sensory analysis are needed to discriminate between healthy and infected or infested plants. It is also consistent with our observations that the application of electronic nose technology should support, guide and optimise the diagnostic techniques traditionally used.

Alternatively, the morphology of wood rot fungal fruiting bodies collected from infected trees can be reviewed. An expert opinion on their behaviour (wood rot type caused) and consequences for risk can be provided. In the absence of fungal fruiting bodies, wood can be examined for the presence of fungi by DNA extraction and amplification of ITS -rDNA by PCR and sequencing of ITS regions.

Schmidt et al. [50] collected wood samples from infected urban trees, extracted total DNA from infected wood, amplified and sequenced the fungus ITS. By comparing the sequences examined with the NCBI gene bank data, the species can be identified. This technique allows accurate and rapid identification of fungi that cause tree rot. However, due to its high workload, it can only be applied to valuable trees or, conversely, to determine if fungal species are not protected by law. However, we would like to focus on detecting soil fungi in forest nurseries as producers of healthy seedlings for future sustainable and diverse forests.

In this article, we do not focus on the effect of so-called resistance fertilisers, to which we can include Actifos. However, it is worth mentioning that the action of ammonium phosphate fertiliser stresses the plants, which has been shown in earlier studies [51] and is now (Figure 7). Its role as a resistance elicitor probably lies in its slightly toxic effect on root systems, especially fine roots. This effect triggers defence processes (synthesis of phenolic compounds, terpenes and sterols) that protect the roots against infections. In the experiment, a concentration of 0.6% was used, which is recommended by the manufacturer of Actifos for ornamental plants. However, this concentration is very low and has not been used successfully in forestry. Much higher concentrations have been used in oak stands, e.g., a 50% concentration of the spray [52] by aerial spraying. The atomizers used in this case allow for a fine droplet size of 250 microns, whereas hand-held sprayers produce droplets twice that size. Therefore, the manufacturer probably recommended a lower compound concentration, which influenced the poorer results in root protection of ash trees. In Australia, similar concentrations of phosphite preparations sprayed from aircraft, close to 50%, were successfully used to protect stands of eucalyptus against *Phytophthora cinnamomi* [53].

In the first phase of investigating the possible use of e-nose in forestry, we would like to focus on distinguishing between diseased and healthy plants, i.e. between infected and pathogen-free and not between stressed and non-stressed plants. This observation is crucial in nurseries where we select material for planting in forest plantations. It is the only way to eliminate diseased seedlings that will carry the disease there if planted in the forest. It should be emphasised that stressed plants, activating defence processes against harmful factors, produce secondary metabolites that the electronic nose can monitor. In this case, we will be able to detect physiological changes before permanent disease symptoms appear. This knowledge will allow early decisions on further management and the development of appropriate countermeasures. Commercial devices such as the Handy PEA are also available for stress testing and can be used to measure chlorophyll fluorescence, which is inversely related to photosynthetic efficiency [54]. However, the stress is temporary, and the plant recovers as soon as the stress ceases. Conversely, suppose an asymptomatic but infected plant is planted under conditions favourable to the pathogen (e.g., full soil saturation with water). In this case, the plant quickly becomes diseased and dies, even though it shows no symptoms in the nursery because the pesticides have masked the full development of the disease. Stress studies must be considered complementary in our case better to understand the predisposition of plants to infection by pathogens and confirm that infection has already occurred when the plant has responded with gene expression and production of resistance proteins. Plants have complex adaptive mechanisms at the cellular and molecular levels. Under stress, plants transcribe and translate heat shock proteins (*HSPs*). *Hsp*s’ genes from distinct plant species respond differently to various types of stress [27,55]. While their response to heat, cold, osmotic, and salt stress is relatively well-studied [56,57], information about their response to pathogen infection is rather scarce. This study revealed an intense response of ash immune mechanisms to *A. gallica* infections by activating the expression of the *Hsp*’s and *Hstf* genes. Influence of *H. fraxineus* and *A. gallica* on *Hsp* ’s has not yet been described. However, other phytopathogenic fungi have an overall effect on the expression of these genes. Analysis of the expression of the *Hsp90* genes in silver birch leaves showed that *Phytophthora plurivora* infection combined with 60% defoliation increased *Hsp90* expression about seven times [58]. *Phytophthora* infestans led to up-regulation of *Hsp70* genes and increased synthesis of *Hsp70* proteins in tomatoes [59] and *Solanum tuberosum* [60], which indicates that it could participate in mediating the disease resistance in plants in response to biotic stresses.

The synthesis of biologically active substances by plants is an effective method to reduce the attack of insects and the spread of pathogens. Biochemical compounds, such as volatile substances (belonging to secondary metabolites produced by the synthesis of terpenes and phenylpropanoid compounds) and growth inhibitors and some hormones are involved in plant defence responses to stress. Plants under the stress of defoliation generally reduce the production of sugars, proteins, starch, lignin, and hemicellulose while increasing the synthesis of secondary metabolites, including phenols, terpenes, and sterols [61].

The above result shows that the sensor signals collected just after the abrupt change of the measurement conditions from clean air to the measured gas conditions exhibit the most significant power of the discrimination between the sample categories. Other authors have reported similar results [62,63,64], when different electronic noses devices and types of odours have been studied. It has also been reported [65,66] measurement technique in "sniffing" mode when frequent changes between studied odour and pure air occur or in the initial time of the sensors’ action.

## 5. Summary

The accidental introduction of the fungus *H. fraxineus* (Hf) from Asia to Europe has caused mass dieback of ash trees, including *F. excelsior*. Infected seedlings and lack of resistance cause shoot death, which favours the development of the root pathogen *Armillaria*. Root damage causes trees to topple over in the forest and the city, where trees grow in limited areas. With the e-nose, we hope to detect the disease early while the trees are still in the nursery. In this way, healthy seedlings can be selected to better survive and develop as a forest-forming tree species, planted in plantations free of the pathogen inoculum. The e-nose still needs to be refined, but initial studies are encouraging, especially as it is the only device that can detect root rot without exposing the roots. *Armillaria* stress increases *Hsp*s gene expression by 2.0 to 2.8 times, while Actifos fertiliser increases *Hsp*s expression by 1.4 to 2.3 times. Simultaneous application of Actifos and *Armillaria* does not alter *Hsp*s expression. Actifos and *Armillaria* increase the expression of a transcription factor that regulates *Hsp*s expression. Actifos has a greater effect on *Hstf* expression (5.8-fold) than *Armillaria* (3.0-fold). Actifos and *Armillaria* have additive effects on *Hstf* expression. Actifos 5.8 + *Armillaria* 3.0 = 8.8-fold, in the experiment 7.1-fold. This shows that both *Armillaria* and Actifos induce stress, but the simultaneous application of Actifos and *Armillaria* does not induce stress in ash trees. Since *Hsp*s expression increases under the influence of *Armillaria*, the trees should be more resistant to *Hymenoscyphus*, provided that *Armillaria* does not destroy their roots so that the wind uproots them. Increases of *Hsp*s in leaves indicate that *Armillaria* is stress factor for *F.excelsior* what is expressed at least in leaves.

## Figures and Tables

**Table 1:** Descriptive statistics for the number of dead shoots and weight of roots dry biomass at the end of the test in each of the studied samples categories. The *p*-value for the Kruskal–Wallis test by ranks. The studied categories of samples: Actifos+*A. gallica* (AG), Control (C), Actifos (A), *A. gallica* (G), Isolated Control (I).

		Number of Shoots					Weight of Roots				
	N	Avg	Min	Max	Std	*p*-Value	Avg	Min	Max	Std	*p*-Value
AG	10	5.4	0	13	4.8	0.27	75	26	199	62	0.0041
C	8	4.4	0	9	3.3	0.38	93	21	494	163	0.00001
A	10	7.3	1	17	4.8	0.62	36	6	74	22	0.54
G	10	7.1	1	12	3.9	0.37	57	8	186	56	0.034
I	10	3.1	0	16	5.3	0.0003	604	27	1072	359	0.45

**Table 2:** Sensor array details in PEN3 electronic nose device, as reported in the menu of options of the e-nose software.

Sensor	Main Gas Targets
W1C	Aromatic organic compounds.
W5S	Very sensitive, broad range sensitivity, reacts to nitrogen oxides, very sensitive to negative signals.
W3C	Ammonia, also used as sensor for aromatic compounds.
W6S	Detects mainly hydrogen gas.
W5C	Alkanes, aromatic compounds, and nonpolar organic compounds.
W1S	Sensitive to methane. A broad range of organic compounds detected.
W1W	Detects inorganic sulfur compounds, e.g., H${}_{2}$S. Also sensitive to many terpenes and sulfur-containing organic compounds.
W2S	Detects alcohol, partially sensitive to aromatic compounds, broad range.
W2W	Aromatic compounds, inorganic sulfur and organic compounds.
W3S	Reacts to high concentrations of methane (very selective) and aliphatic organic compounds.

**Table 3:** Parameters of PEN3 electronic nose operations.

Sampling Interval	1 s
Pre sampling time	5 s
Measurement Time	120 s
Flush Time	300 s
Chamber Flow	7.7 mL/min
Temperature	25 °C
Humidity	60%

**Table 4:** List of primers. Genes ID according to https://hardwoodgenomics.org (accessed on 1 June 2021).

Gene	ID	Primes Name	Sequence
*Hsp17*	Fraxinus_pennsylvanica_120313_comp43352_c0_seq1	FrHsp17f	GGTGGACAAGCCGGTAGTTA
		FrHsp17r	ACGCAAATCTTCACCTTTGG
*Hsp70*	Fraxinus_pennsylvanica_120313_comp60882_c0_seq2	FrHsp70f	CTGGGGAGGAAAGATCATCA
		FrHsp70r	CAACTTCTGGTTTCGGGTGT
*Hsp90*	Fraxinus_pennsylvanica_120313_comp64929_c0_seq2	FrHsp90f	AGCATGAAGCCACTCTCCAT
		FrHsp90r	CGAAATTAACCCGAGACACC
*Hstf*	Fraxinus_pennsylvanica_120313_comp62864_c0_seq1	FrHsff	TGGTCCCAAGATTGAGGAAG
		FrHsff	AGGATCATGCATTTCCGAAG
*Tub*	Fraxinus_pennsylvanica_120313_comp63421_c0_seq2	FrTubf	TGCATGTGGAAGAAATGGAA
		FrTubr	AGGGGAAGAATGGAAGAGGA

## Data Availability

The data presented in this study are available from the corresponding author.

## Supplementary Materials

The following are available online at https://www.mdpi.com/article/10.3390/pathogens10111359/s1, Figure S1: Whole experimental design; Figure S2: Experimental plan from the Chojnów Forestry District (photo taken 25 September 2019 by Artur Pacia); Figure S3: Sensors responses as conductance normalized by the baseline value (G/G0), for measurements of roots samples of Actifos (A) category; Figure S4: Sensors responses as conductance normalized by the baseline value (G/G0), for measurements of roots samples of Actifos+Gallica (AG) category; Figure S5: Sensors responses as conductance normalized by the baseline value (G/G0), for measurements of roots samples of Control (C) category; Figure S6: Sensors responses as conductance normalized by the baseline value (G/G0), for measurements of roots samples of Gallica (G) category; Figure S7: Distribution of modelling feature extracted from the sensor response characteristics (integral/sum of the response data) for measurements of soil samples. The exact value of the y-axis for various sensors has no physical interpretation, thus is presented only in arbitrary units. The studied categories of samples: Actifos (A), Actifos+Gallica (AG), Gallica (G), Isolated Control (I), Control (C) are plotted in the x-axis. In these boxplots, the horizontal line inside the box represents the sample median, the box area spans from the 1st to the 3rd quantile, the whiskers span from Q1 − 1.5*IQR to Q3 + 1.5*IQR (IQR-interquantile range). In this plot, we overlay all data points presented by dots, which allow us to see outliers and the whole distribution of data.
