# Supplementary material for: Advances in the Detection of Emerging Tree Diseases by Measurements of VOCs and HSPs Gene Expression, Application to Ash Dieback Caused by Hymenoscyphus fraxineus
Source: Pathogens. 2021 Oct 21;10(11):1359. doi: 10.3390/pathogens10111359 (PMC8622506; doi:10.3390/pathogens10111359)
Supplement: Supplementary file 1 [file pathogens-10-01359-s001.zip › pathogens-1349106-supplementary.pdf]

Supplementary Materials: Advances in the Detection of Emerging Tree Diseases by Measurements of VOCs and HSP gene expression, Application to Ash Dieback Caused by *Hymenoscyphus fraxineus*

Piotr Borowik, Tomasz Oszako, Tadeusz Malewski, Zuzanna Zwierzyńska, Leszek Adamowicz, Rafał Tarakowski, Sławomir Ślusarski, Justyna Nowakowska

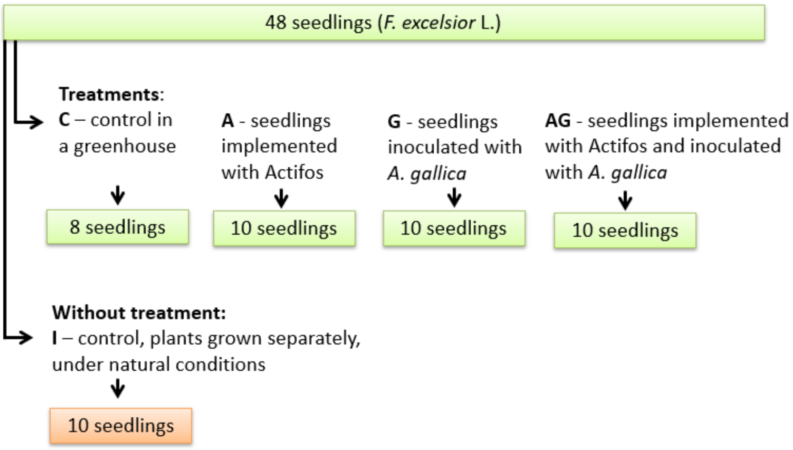

Figure S1. Whole experimental design.

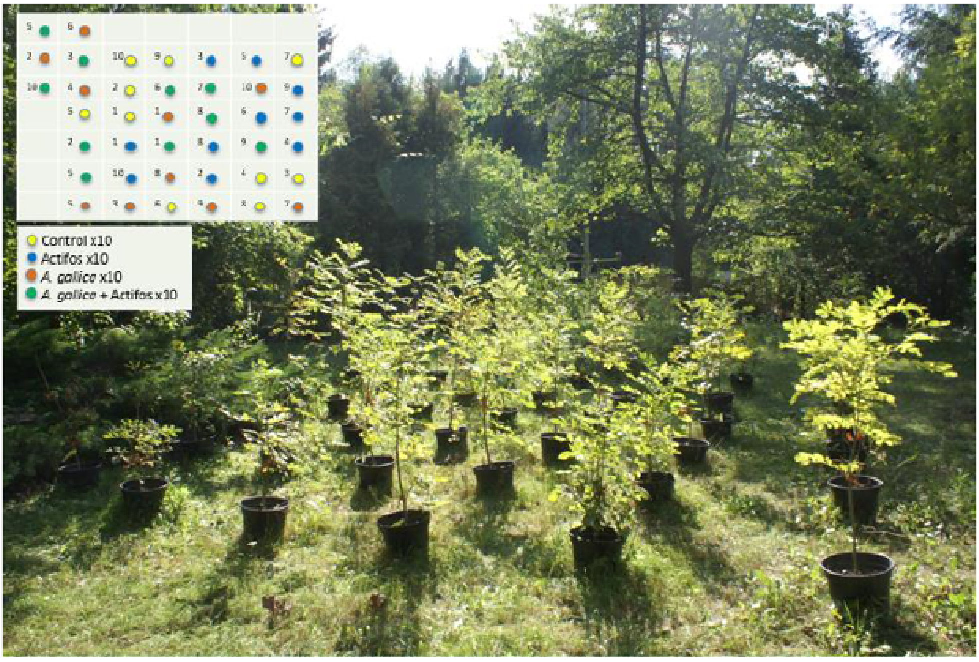

Figure S2. Experimental plan from the Chojnów Forestry District (photo taken 25.09.2019 by Artur Pacia).

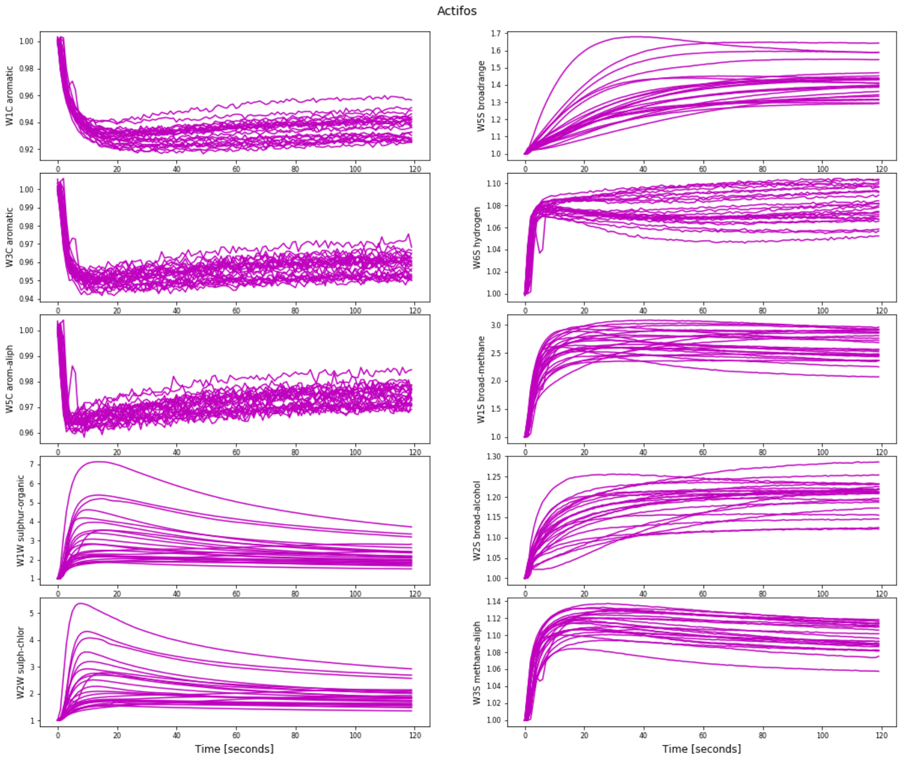

Figure S3. Sensors responses as conductance normalized by the baseline value (G/G_0), for measurements of roots samples of Actifos (A) category.

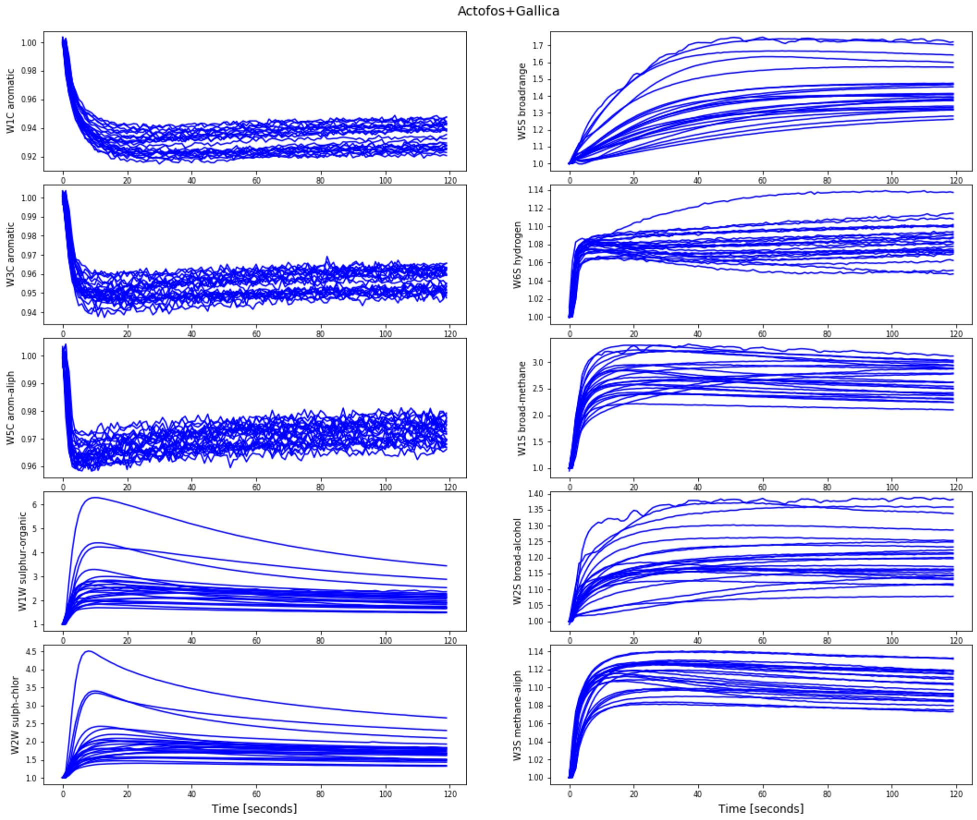

Figure S4. Sensors responses as conductance normalized by the baseline value (G/G_0), for measurements of roots samples of Actifos+*Gallica* (AG) category.

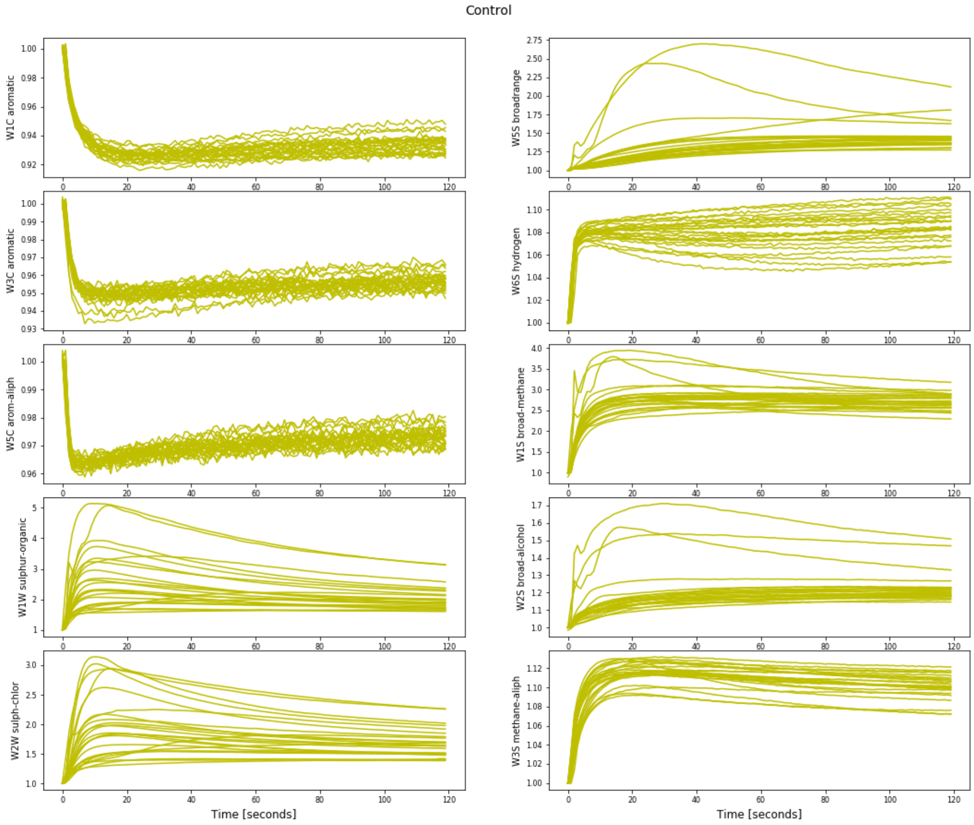

Figure S5. Sensors responses as conductance normalized by the baseline value (G/G_0), for measurements of roots samples of Control (C) category.

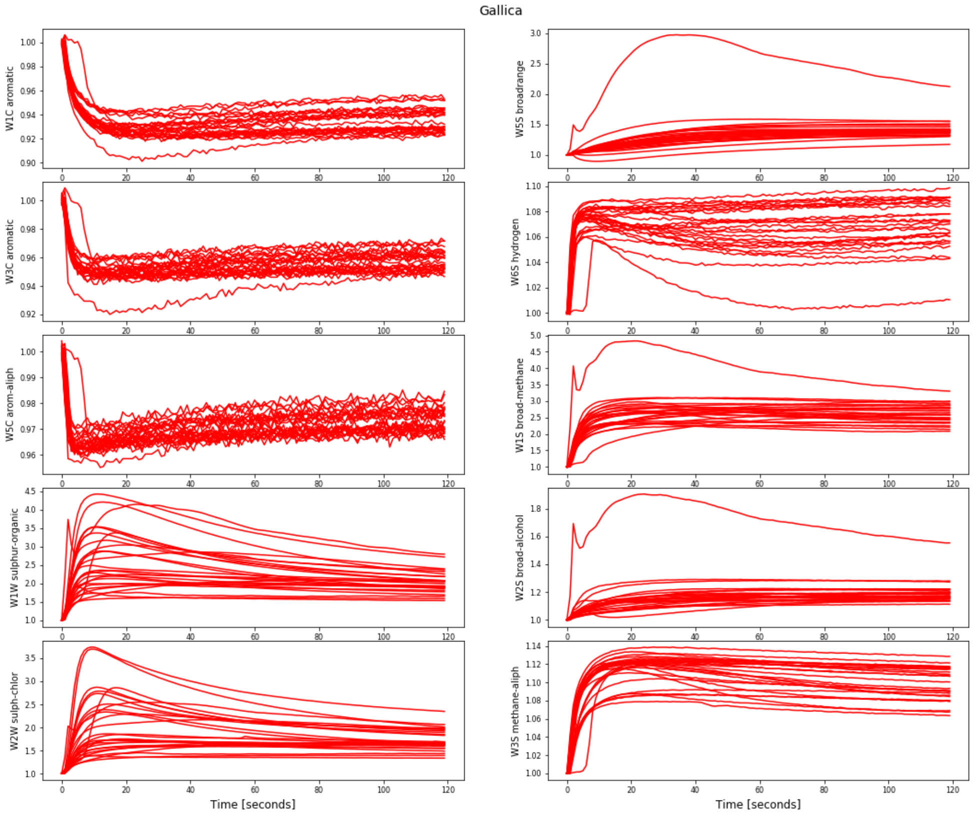

Figure S6. Sensors responses as conductance normalized by the baseline value (G/G_0), for measurements of roots samples of *Gallica* (G) category.

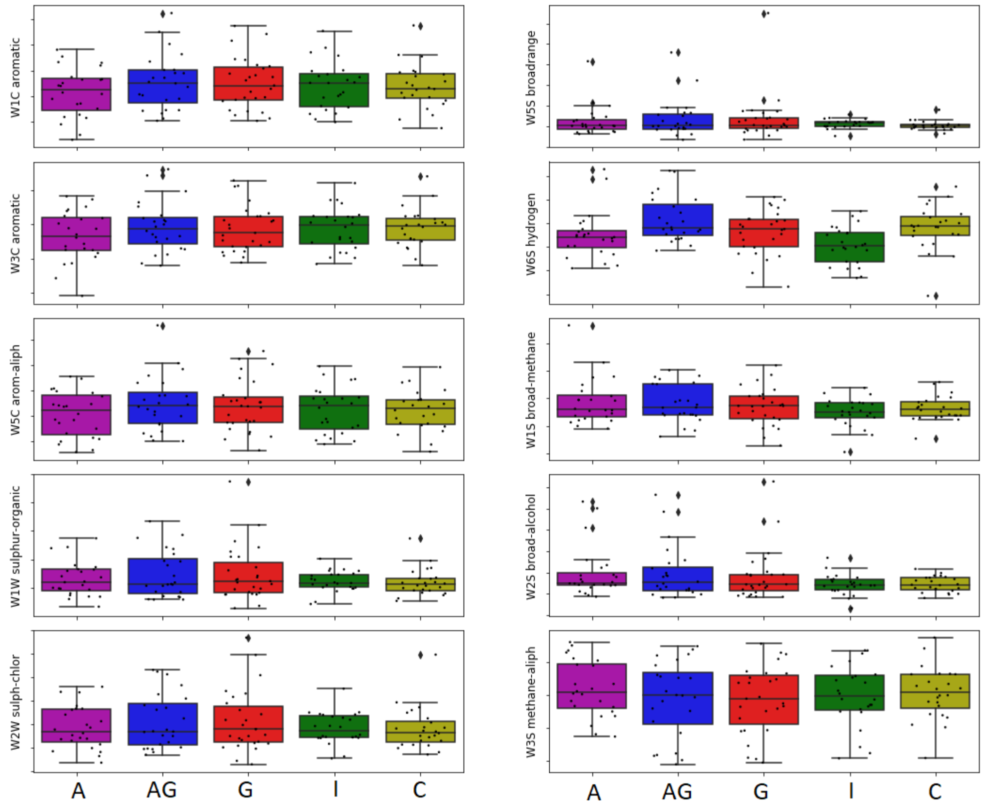

Figure S7. Distribution of modelling feature extracted from the sensor response characteristics (integral/sum of the response data) for measurements of soil samples. The exact value of the y-axis for various sensors has no physical interpretation, thus is presented only in arbitrary units. The studied categories of samples: Actifos (A), Actifos+*Gallica* (AG), *Gallica* (G), Isolated Control (I), Control (C) are plotted in the x-axis. In these boxplots, the horizontal line inside the box represents the sample median, the box area spans from the 1st to the 3rd quantile, the whiskers span from Q1-1.5*IQR to Q3+1.5*IQR (IQR - interquantile range). In this plot, we overlay all data points presented by dots, which allow us to see outliers and the whole distribution of data.
